# Confamiliar transferability of simple sequence repeat (SSR) markers from cotton (*Gossypium hirsutum* L.) and jute (*Corchorus olitorius* L.) to twenty two Malvaceous species

**DOI:** 10.1007/s13205-016-0392-z

**Published:** 2016-02-15

**Authors:** Pratik Satya, Pramod Kumar Paswan, Swagata Ghosh, Snehalata Majumdar, Nasim Ali

**Affiliations:** 1 Central Research Institute for Jute and Allied Fibres, Barrackpore, Kolkata 700120 India; 2 Ramakrishna Mission Vivekananda University, Narendrapur, West Bengal India

**Keywords:** SSR, Confamiliar transferability, Cotton, Jute, Malvaceae, Taxonomic groups

## Abstract

Cross-species transferability is a quick and economic method to enrich SSR database, particularly for minor crops where little genomic information is available. However, transferability of SSR markers varies greatly between species, genera and families of plant species. We assessed confamiliar transferability of SSR markers from cotton (*Gossypium hirsutum*) and jute (*Corchorus olitorius*) to 22 species distributed in different taxonomic groups of Malvaceae. All the species selected were potential industrial crop species having little or no genomic resources or SSR database. Of the 14 cotton SSR loci tested, 13 (92.86 %) amplified in *G. arboreum* and 71.43 % exhibited cross-genera transferability. Nine out of 11 jute SSRs (81.81 %) showed cross-transferability across genera. SSRs from both the species exhibited high polymorphism and resolving power in other species. The correlation between transferability of cotton and jute SSRs were highly significant (*r* = 0.813). The difference in transferability among species was also significant for both the marker groups. High transferability was observed at genus, tribe and subfamily level. At tribe level, transferability of jute SSRs (41.04 %) was higher than that of cotton SSRs (33.74 %). The tribe Byttnerieae exhibited highest SSR transferability (48.7 %). The high level of cross-genera transferability (>50 %) in ten species of Malvaceae, where no SSR resource is available, calls for large scale transferability testing from the enriched SSR databases of cotton and jute.

## Introduction

Microsatellites or simple sequence repeats (SSRs) are tandem repeats of short sequences (1–6 nucleotides) distributed throughout genomes, which can be identified through simple polymerase chain reaction (Morgante and Olivieri 1993). SSR markers are locus specific, multi-allelic, codominant and are highly polymorphic in plant and animal species (Wang et al. 2009). By virtue of their locus specificity and high polymorphism, SSRs are considered ideal DNA marker systems for evolutionary analysis, phylogeny reconstruction, genetic mapping and molecular breeding (Kalia et al. 2011). But the primary bottleneck of using SSRs in molecular research is the cost and effort required for designing of specific primers for amplification of locus specific SSRs, which involves sequencing of targeted genomic regions (Squirrell et al. 2003). This has limited the use of SSR markers primarily to species with enriched genomic resources. An alternate useful approach is to search for SSR markers transferable to closely related species or genera, which brings down the cost and time required for SSR development, particularly in species with limited or no genomic information (Peakall et al. 1998). Sequence data obtained from several crop species indicate sufficient homology between genomes in the regions harbouring microsatellites. Recently, comparative genomics in *Brassica* have shown that microsatellite characteristics in related species are highly similar (Shi et al. 2014). Thus, primer pairs designed on the basis of sequence of one species could be used to develop SSR markers for other related species. Such cross-amplification has been successfully utilized in many crops and their wild relatives for genetic differentiation and evolutionary studies (Sudheer et al. 2011; Tabbasam et al. 2014).

Cross-transferability of SSRs is often more successful between closely related species (Peakall et al. 1998; Balachandran et al. 2013). Limited information is available on confamiliar transferability of SSR markers in plants. Transfer rates of SSRs between genera is approximately 10 % in Eudicots (Barbará et al. 2007), although high transferability between distant genera was observed in some cases (Gutierrez et al. 2005; Rai et al. 2013). But a systematic study to assess efficiency of transferability of SSR markers across different taxonomic orders in plants is limited.

The family Malvaceae harbours over 2300 diverse species belonging to 200 genera, of which only few species, cotton (*Gossypium hirsutum* L.), jute (*Corchorus olitorius* L.) and cocoa (*Theobroma cacao*) are industrially important crop species. Cotton is very rich in genomic resources, where more than 5000 SSR markers have been developed (Xiao et al. 2009). Similarly 2469 SSR markers have been developed in jute (Mir et al. 2009). But genomic resources are poor in other Malvaceous species. A good number of potential industrial crops and semi-domesticated species belong to Malvaceae, such as fibre crops like *Hibiscus cannabinus* (kenaf), *Hibiscus acetosella* (false roselle), *H. vitifolius*, *Abutilon indicum* (Indian mallow), *Abroma augustum* (Devil’s cotton), *Sida spp.*, *Urena lobata*; ornamental crops like *H. rosa*-*sinensis* (China rose), *Alcea rosea* (hollyhock), *Malvaviscus arboreus* (Turk’s Cap), *H. mutabilis* (cotton rosemallow); and medicinally important species like *Wissadula periplocifolia* and *Abelmoschus moschatus*. Genomic resources are scanty in these species; thus cross-species transfer of SSRs from cotton and jute may be better option to develop SSR database in these Malvaceous species. A few recent reports indicate successful transferability of SSRs within same genus in Malvaceae (Bruna et al. 2009; Tabbasam et al. 2014), but these studies are limited to closely related species. We have earlier observed successful intergeneric amplification of genomic SSRs from jute in *Hibiscus cannabinus* and related wild species (Satya et al. 2013), which indicates the possibility of high SSR transferability in Malvaceae across genera. In the present study, we targeted amplification of SSR markers designed from cotton and jute in diverse Malvaceous genera to compare confamiliar transferability of SSR markers across different taxonomic levels, and also to investigate possibility of developing SSR resources in some minor crop species of Malvaceae through cross-transferability.

## Materials and methods

### Plant materials and DNA isolation

We studied cross species transferability of SSR markers from cotton (*G. hirsutum*) and jute (*C. olitorius*) in 22 Malvceous species belonging to 12 genera. Eight *Hibiscus* species (*H. cannabinus, H. surattensis, H. acetosella, H. rosa*-*sinensis, H. schizopetalus, H. vitifolius, H. mutabilis* and a synthetic *H. cannabinus x H. surattensis* allopolyploid) and one representative species each from *Althea* (*A. rosea*), *Abutilon* (*A. indicum*)*, Abroma* (*A. augustum*) and Urena (*U. lobata*) were obtained from Central Research Institute for Jute and Allied Fibres, Barrackpore, West Bengal, India (22°45′35″N and 88°25′36″E). Three *Sida* species (*S. cordifolia, S. acuta, S. rhombifolia*), two *Abelmoschus* species (*A. esculentum* and *A. moschatus*) and one representative species each from *Gossypium* (*G. arboretum*), *Malvaviscus* (*M. arboreus*)*, Malvastrum* (*M. auratiacum*)*, Pavonia* (*P. odorata*) *and Wissadula* (*W. periplocifolia*) were collected from medicinal plant garden of Ramakrishna Mission Vivekananda University, Narendrapur, West Bengal (22°26′18″N and 88°24′1.4″E). In addition, cross-genus transferability of cotton SSRs was also tested in *C. olitorius*. We did not test transferability of jute SSRs in cotton as cotton SSR database is already enriched. All the species were grown and identified through morphological characterization. Genomic DNA was extracted from leaf samples of single representative plant of each species by CTAB method (Doyle and Doyle 1990) with modifications (Satya et al. 2013). Leaf tissues were extracted in extraction buffer containing CTAB (2 %), Tris–HCl (100 mM), NaCl (1.4 M), EDTA (20 mM) and 0.1 M β-mercaptoethanol, and incubated at 60 °C for 1 h. One ml of extract was mixed gently with equal volume of dichloromethane and centrifuged at 14,000×*g* for 15 min. The supernatant was mixed with isopropanol (1 ml) and centrifuged at 14,000×*g* for 5 min to get the DNA pellet. The pellet was kept in 100 % ethanol at −20 °C for 24 h, washed with 70 % ethanol, air dried and stored in Tris–EDTA (10 mM) at −20 °C for further analysis. The quality of the extracted DNA was checked by A_260_/A_280_ ratio using a spectrophotometer (Biospectrometer, Eppendorf, Germany).

**Table 1:** SSR primers used for cross-amplification in Malvaceae

Species	Primer	Primer sequence		*T*_m_ (°C)	No. of alleles	Allele size (bp)	PIC	*R*_p_
Cotton	C2-0004C	F	TTTGTTTTGCGTTCCTTTA	49.2	2	201	0.99^a^	0.17
		R	TCCGACAATGCCTTACAAG					
	C2-0005B	F	CCCCATTCCTACTCATCC	51.0	10	306	0.76	3.04
		R	CACAGAAAGGTGCTCATGC					
	CGR5501	F	TCTCTCTTGCTGGTCACGAA	53.1	6	145–166	0.95	0.96
		R	TGCCAAATACCCAAATCCAT					
	CGR5503	F	GCTGCTTCCATGCCATTATT	57.5	3	111–127	0.44	2.26
		R	GGGTCGCTTTGTAAGTGAATG					
	CGR5506	F	CAGCAACCACAATTCGATCA	57.5	5	156	0.66	2.43
		R	GAAGTTGCTGTTGGGAAGGA					
	CGR5508	F	CAACTTTCCGAGCTGGATTC	56.1	4	680	0.88	1.30
		R	TGATCGAGGAATGAAAGCAA					
	CGR5645	F	GAGCGGAGAGTCCGGTTT	54.5	2	134	0.99^a^	0.17
		R	CCCAAACGAATCAAAGATGG					
	DC30003	F	AGGAGGGAAAGAGTGGTG	48.0	9	256	0.73	2.87
		R	CCTCCTCACATCCAATCA					
	DC30005	F	ATGAGAAACGGTGTCGAA	53.2	6	188	0.88	1.48
		R	TTGACCGAATACTCCCCT					
	DPL0840	F	GAGTCGTTGCCGCTGTTTA	55.2	5	152–182	0.42	2.61
		R	GCTACGACTCGATGTTACGG					
	DPL 0848	F	AACCCAACCATCTTCACTGC	55.0	6	255	0.99	0.52
		R	TTGGTTTCCGATAGCCATAA					
	SHIN0733	F	GCTTTGCCTTCGGTTCATT	56.5	3	208	0.95	0.26
		R	GGACTTCGCTTTATGAATGCTT					
	SHIN0745	F	GCACCGAGTCTCCTATGCTC	60.3	4	162	0.94	0.96
		R	GGACCCTCAAACTTGTATTACACT					
Jute	MJM 006	F	ACGTTTAGCAACTGATATTGG	54.9	8	143	0.90	1.55
		R	ACTTACAGCGGTTACATCATT					
	MJM 211	F	ACGACAATCAATACGACAATC	54.5	4	302	0.84	1.45
		R	ATTCAGGCTTGATAACAGTGA					
	MJM 472	F	CCATTCGTAGCATTAAAGTTTGC	55.5	2	177	0.53	1.45
		R	GATTGTGTGCAAACACGAGAG					
	MJM 536	F	GTAGCCAAGTCTGCTTCCTGA	56.0	2	316	0.99^b^	0.09
		R	TAGGTCACGAGAAGAGCGAAG					
	MJM 563	F	CTTGGTTGTGGTGGTTGAACT	55.5	5	318	0.87	1.36
		R	AAACCCACCATAGTTGTGTGC					
	MJM 609	F	TCAAATCCAAGCACCCATAAA	54.2	8	334	0.76	2.55
		R	AGAATTTGCGAAGTGGGCTAT					
	MJM 618	F	CGTTATCAAGCAAATCCAACC	54.5	8	305	0.79	2.36
		R	CATCTGGTGACTGCTTCGTCT					
	MJM 623	F	TTCTGCAGTTGTCTCCCTGTT	60.0	8	319	0.66	2.36
		R	ACGAGAAGACACAGTGGTGCT					
	MJM 634	F	GGAGAATATAAGGCCGCGTAG	62.2	3	110	0.83	1.00
		R	CAGCGGTGTAAGGCTCTCTC					

*T*_m_ Melting temperature, *PIC* polymorphism information content, *R*p resolving power

^a^Amplified only in *G. arboreum*

^b^Amplified only in *H. rosa*-*sinensis*

### Microsatellite markers and PCR analysis

A total of 14 SSRs specific to *G. hirsutum* (Xiao et al. 2009) and 11 SSRs specific to *C. olitorius* (Mir et al. 2009) were used to examine cross-genera transferability. The primers were synthesized from Metabion, Germany. The PCR reaction mixture (20 µl) contained 20 ng of template DNA, 0.4 µl of dNTPs (0.2 mM) (Invitrogen, USA), 2 µl of 50 mM MgCl_2_, 0.4 µl of each primer (10 µM), 1 U of *Taq DNA polymerase* (Invitrogen, USA) and 2 µl of 1× PCR buffer. The samples were amplified in a thermal cycler (Mastercycler Nexus Gradient, Eppendorf, Germany) with an initial denaturation at 94 °C for 5 min; 30 cycles of denaturation at 94 °C for 1 min, primer annealing at specific annealing temperature for 1 min and primer extension at 72 °C for 1 min; final extension at 72 °C for 8 min. The annealing temperature was initially determined as 2 °C lower than melting temperature (*T*_m_) and varied for obtaining optimal annealing temperature for obtaining reproducible amplification (Table 1). PCR products were separated using 6 % polyacrylamide gel electrophoresis using 100 bp DNA ladder (Invitrogen, USA) as standard. Amplification patterns were photographed in a gel documentation system (Bio-Rad, USA).

### Data analysis

Each analysis was replicated five times and signals were considered positive if 80 % of the samples exhibited PCR amplification. Based on signal intensity, the amplicons were classified in four classes following Kuleung et al. (2004) and Rai et al. (2013). Amplicons having moderate to strong signals were considered for further analysis. Cross-genera transferability of each SSR was determined by the percentage of genera amplifying the marker. Cross-genera transferability of all the SSR markers in a genus was calculated as the percentage of amplified SSRs in that genus. Significance of the difference in transferability of SSRs was tested by Student’s *t* test. We also estimated the correlation between transferability of jute and cotton SSRs and tested the significance of the correlation by *t* test. The polymorphic information content (PIC) of each SSR marker was calculated using the formula: PIC = 1−∑ (*p*_*i*_)^2^ where *p*_*i*_ is the frequency of the *i*th allele. Marker resolving power was determined from band informativeness (*I*_b_) as suggested by Anderson et al. (1993).

## Results and discussion

The present study is the first report on confamiliar transferability of SSR markers in Malvaceae. The study was carried out to determine the transferability of jute and cotton microsatellites to 22 Malvaceous genera. Out of 14 cotton SSRs 13 markers produced a total of 66 alleles in 23 species (including *C. olitorius*) with a size range of 112–923 bp (Average 336 bp). Four SSR markers, C2-005B, DC300003, DPL0840 and CGR5503 exhibited more than 50 % transferability in these species. The number of alleles amplified by cotton SSRs ranged from 2 to 10 with an average of 5.08 alleles per marker (Table 1). The PIC values for the SSR markers varied from 0.42 for DPL0840 to 0.99 for DPL0848, with an average of 0.78. Marker C2-0005B exhibited highest resolving power (3.04), while resolving power per allele was highest for CGR5503 (0.75) (Table 1).

Significant differences among species was observed for cotton SSR transferability (*t*_19_ = 5.84, *P* < 0.001). Of the 14 *G. hirsutum* SSR markers 13 were amplified in sister taxa *G. arboreum* (92.86 % transferability) producing a total of 35 alleles. Since the tetraploid *G. hirsutum* genome (*AABB*) originated from diploid *AA* genome of *G. arboreum*, high SSR transferability is expected between these two species. Three SSRs C2-004C, CGR5645 and SHIN-0733 failed to amplify in other genera. Among the 12 genera, transferability was highest in *Abutilon* (57.14 %) and *Malvaviscus* (57.14 %). No SSR was amplified in *Malvastrum* and *Urena*. Transferability of cotton SSRs was higher in *Corchorus* (50.0 %) than in *Hibiscus* (40.18 %). At the species level, highest transferability was exhibited by *H. schizopetalus* (64.29 %). The species *S. rhombifolia*, *A. moschatus*, *M. auranticum* and *U. lobata* did not show any reproducible amplification.

Nine SSR markers from jute exhibited successful cross-genera amplification, whereas two SSRs failed to amplify in other Malvaceous species. A total of 48 alleles were amplified by these SSRs with an average of 5.3 alleles per marker (Table 1). The allele size ranged from 104 to 759 bp with an average of 331 bp. Average PIC value for jute SSRs was 0.80 (range 0.53–0.99). Highest *R*_p_ was exhibited by MJM 618 and MJM 623 (2.36).

The resolving power of a marker is an indication of the utility of the markers in assessing genetic differentiation and diversity. A resolving power of greater than one is considered more informative (Azevedo et al. 2012). Out of 23 markers used in the study, 15 markers exhibited a *R*_p_ value of ≥1.0 (Table 1). The average *R*_p_ of cotton (1.46) and jute (1.58) SSRs indicated that both groups of SSRs are useful for genetic discrimination and diversity studies, though more number of jute SSRs (88.9 %) exhibited resolving power of ≥1.0 compared to cotton SSRs (40 %). The resolving powers of the cotton and jute SSRs observed in Malvaceae are similar to our earlier findings in *Hibiscus* (Satya et al. 2013) and also to other cross-transferability studies, such as *Vigna angularis* SSRs in *Vigna radiata* (Singh et al. 2014) and pearl millet SSRs in *Pennisetum purpureum* (Azevedo et al. 2012).

**Fig. 1:**
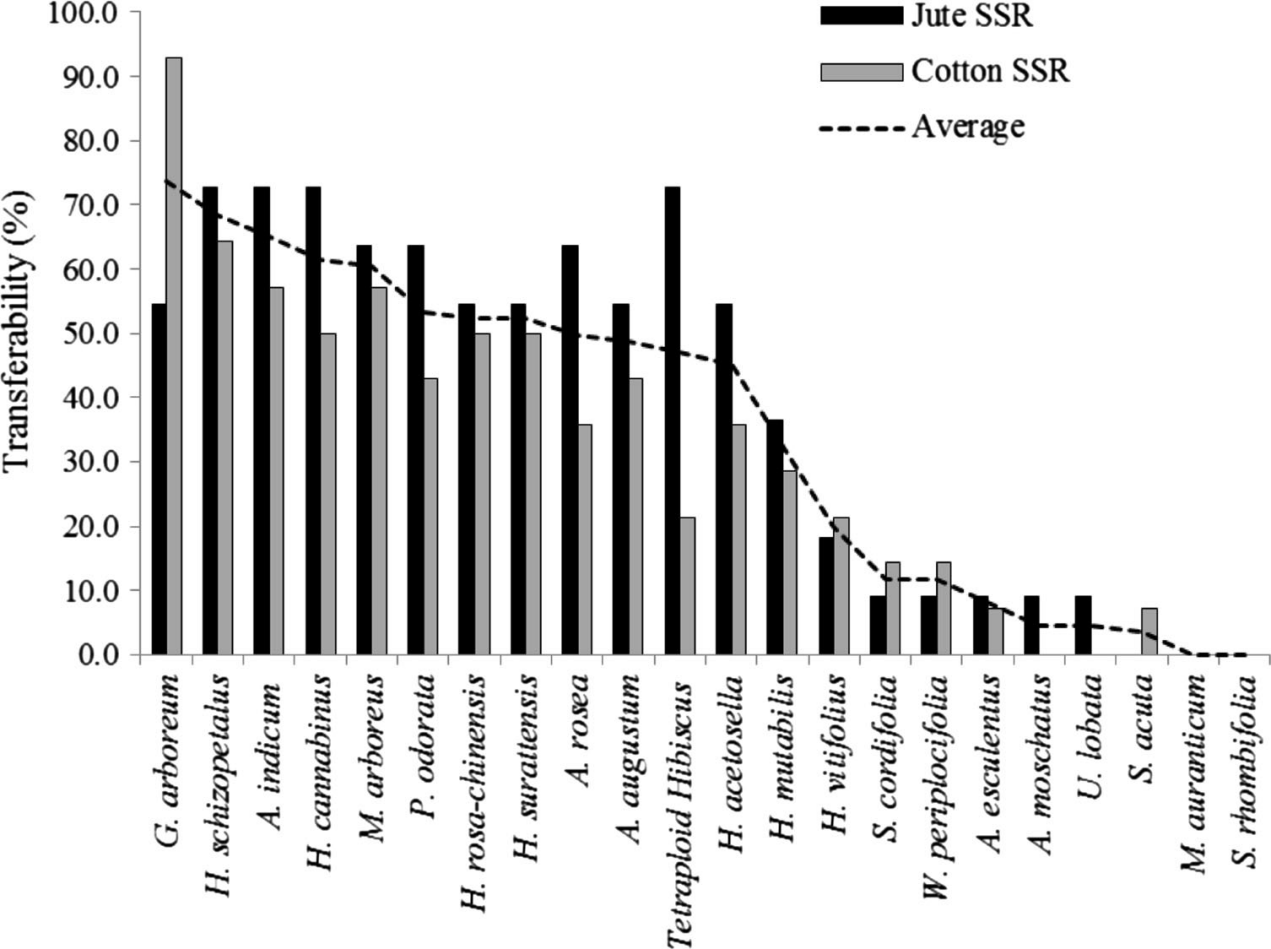
Comparative confamiliar transferability of jute and cotton SSR markers

Average cross-genera transferability of Jute SSRs was higher (38.84 %) than that of cotton SSRs (32.30 %; Fig. 1). Cross-genera transferability was highest in *Abutilon* (72.73 %) followed by *Malvaviscus* and *Pavonia* (63.64 %). The jute SSRs showed lower transferability to *Gossypium* (54.55 %) than cotton SSRs. The SSRs failed to amplify in *S. rhombifolia*, *S. acuta* and *M. aurantiacum*. At the species level, significant difference was observed for SSR cross-amplification (*t*_19_ = 6.40, *P* < 0.0001). Highest transferability was observed in *H. cannabinus*, *H. schizopetalus* and *A. indicum* (72.73 %). We found a significant positive correlation between the cross-species transferability of cotton and jute SSR markers (*r* = 0.813, *P* < 0.0001), which indicate a similar pattern of transferability of these two groups of SSRs in Malvaceae.

Peakall et al. (1998) suggested that cross-transferability of SSRs within a genus can vary from 50 to 100 %, while transferability across genera is usually below 50 %. In the present study, more than 50 % transferability was observed in ten Malvaceous species from different taxonomic groups (Fig. 1). The findings are in accordance with earlier reports on cross-genera transferability, for instance from mulberry to fig and jackfruit (45.74–71.80 %; Balachandran et al. 2013), *Psidium guajava* to other Myrtaceae species (60.8–78.2 %; Rai et al. 2013), tall fescue to several grasses (47.8–66.2 %; Saha et al. 2006), *C. olitorius* to *Hibiscus* *spp*. (48 %; Satya et al. 2013) and *Litchi chinensis* to *Blighia sapida* (58 %) (Ekue et al. 2009).

The thirteen genera in which intergeneric transferability was tested belonged to five tribes, Byttnerieae (*Abroma*), Gossypieae (*Gossypium*), Hibisceae (*Abelmoschus, Hibiscus, Malvaviscus, Pavonia, Urena*), Malveae (*Abutilon, Alcea, Malvastrum, Sida, Wissadula*) and Sparmanniaceae (*Corchorus*) (Stevens 2001). Of these, cross-transferability was successful from Gossypieae and Sparmanniaceae to all the other tribes, establishing high confamiliar transferability of SSRs within Malvaceae. The tribe Byttnerieae exhibited higher cross transferability (48.7 %) than other two tribes Hibisceae (39.21 %) and Malveae (20.22 %). At higher taxonomic level, transferability was also successful from subfamily Grewoideae (containing *C. olitorius*) to other subfamilies Malvoideae (38.09 %) and Byttnerioideae (54.54 %) and also from Malvoideae (containing *G. hirsutum*) to Grewoideae (50.0 %) and Byttnerioideae (42.86 %; Table 2). The extent of transferability, however, did not match well with subfamily classification, since cotton SSRs did not amplify well in *Abelmoschus, Malvastrum, Sida and Urena*, all of which belong to same subfamily (Malvoideae) as cotton. Under Malvoideae cotton belongs to a different tribe Gossypieae, while *Abelmoschus* and *Urena* belong to Hibisceae and *Malvastrum and Sida* belong to Malveae. The other members of Hibsiceae excluding genus *Abelmoschus* exhibited good cross transferability from cotton (42.14 %) and jute (56.36 %), indicating cross-tribe transferability may not be low from Gossypieae and Sparmanniaceae to Hibisceae. The two *Abelmoschus* species included in the study, *A. esculentus* (2*n* = 108−144) and *A. moschatus* (2*n* = 72) bear large genome distributed over many chromosomes (Hamon and van Sloten 1995). *A. esculentus* is an allopolyploid with variable level of ploidy. These factors might have limited intergeneric transferability of SSRs in *Abelmoschus*, but considerable inter-tribe transferability can be realized in Hibisceae.

**Table 2:** Transferability of SSR markers across tribes and subfamilies in Malvaceae

From	To	Transferability (%)
Sparmanniaceae	Hibisceae	32.97
	Byttnerieae	54.55
	Malveae	22.08
	Gossypieae	54.55
Gossypieae	Hibisceae	40.00
	Byttnerieae	42.86
	Malveae	18.37
Grewoideae	Malvoideae	38.09
	Byttnerioideae	54.55
Malvoideae	Byttnerioideae	42.86
	Grewoideae	50.00

Given the high number of SSRs discovered in cotton and jute, confamiliar transferability is more rapid and economic option to develop a robust SSR database in these species. Confamiliar transferability of SSR markers from cotton and jute will benefit molecular genetic studies, species conservation, ecological studies and genetic improvement. The present study did not find good prospects for transferability of SSR markers in *Abelmoschus*, *Malvastrum*, *Sida* or *Urena* from cotton and jute. Although more SSRs need to be screened for comprehensive result, the trend of amplification of SSR markers from two different species indicates transferability of SSR markers may be less successful in these species. Development of SSRs from mining sequence information would be more appropriate for *Abelmoschus*, *Malvastrum*, *Sida* and *Urena*.

The present analysis stands to support that genomic SSRs are transferable across genera, tribes and subfamilies within Malvaceae. The report can serve as a guideline for adopting different approaches of SSR marker development in these species/genera, either by cross-amplification or by sequence mining. As most of the Malvaceous species do not have genomic or EST sequence information, we suggest that 50 % transferability may be optimal for targeting cross-genera amplification. Considering high number of SSR markers in cotton and jute, a transferability of 30 % may also be acceptable to build an initial SSR pool for genomic researches in species where sequence based SSR development may be problematic due to polyploidy or large genome size.
